# Evaluation of pediatric diabetes mellitus after SARS-CoV-2 infection: A long-term prospective case series

**DOI:** 10.1016/j.clinsp.2023.100299

**Published:** 2023-11-20

**Authors:** Thais T. Fink, Ana P.M. Canton, Maria Fernanda B. Pereira, Vera Bain, Olívia Matsuo, Camilla Astley, Heloisa H.S. Marques, Simone Correa-Silva, Marilia M. Montenegro, Patricia Palmeira, Marlene P. Garanito, Alberto J.S. Duarte, Magda Carneiro-Sampaio, Ana C. Latronico, Clovis A. Silva

**Affiliations:** Instituto da Criança, Universidade de São Paulo (USP), São Paulo, SP, Brazil; Hospitas das Clínicas, Faculdade de Medicina da Universidade de São Paulo (FMUSP), São Paulo, SP, Brazil; Instituto da Criança, Universidade de São Paulo (USP), São Paulo, SP, Brazil; Hospitas das Clínicas, Faculdade de Medicina da Universidade de São Paulo (FMUSP), São Paulo, SP, Brazil; Pediatric Department, Faculdade de Medicina da Universidade de São Paulo (FMUSP), São Paulo, SP, Brazil

*Dear Editor,*

Longitudinal studies have revealed that signs and symptoms of pediatric Coronavirus Disease 2019 (COVID-19) can persist and cause disability after infection. Building upon this insight, we established a new outpatient clinic overseen by a multidisciplinary and multi-professional team. This clinic aims to prospectively assess pediatric COVID-19 survivors at our medical center, which is affiliated with a tertiary University Hospital known as a national research reference and the largest Hospital Complex in Latin America.^1^

We recently reported that 43% of children and adolescents exhibited at least one persistent symptom during their initial follow-up visit post-infection. Notably, 23% of these cases were classified as long COVID-19, characterized by the presence of at least one symptom lasting for three months or more. These symptoms included headache (19%), fatigue (9%), dyspnea (8%), and difficulties with concentration (4%).^1^ Furthermore, aside from these long-term symptoms, emerging studies have indicated that Severe Acute Respiratory Syndrome Coronavirus 2 (SARS-CoV-2) infection may also precipitate the onset of new endocrine disorders, including various forms of diabetes mellitus (type 1, type 2, or other diabetes mellitus).^2^, ^3^, ^4^, ^5^

Based on this evidence, we evaluated new-onset diabetes mellitus diagnosis after SARS-CoV-2 infection in our patients. For this purpose, fasting blood glucose and glycated hemoglobin (A1C) tests were systematically analyzed in 53 symptomatic pediatric laboratory-confirmed COVID-19 patients after first and second follow-up visits after SARS-CoV-2 infection. The median duration between SARS-CoV-2 infection and longitudinal follow-up analysis was 4.4 months at the first visit (0.8‒10.7 months) and 12.35 months at the second visit (9.3‒22.5 months). These laboratory exams were also investigated in a control group of 52 children and adolescents, both negative real-time Reverse Transcription-Polymerase Chain Reaction (real-time RT-PCR) for SARS-CoV-2 and antibody-based SARS-CoV-2 analysis and were balanced with pediatric laboratory-confirmed COVID-19 patients by similar age, sex, ethnicity, socio-economic and anthropometric data, and previous pediatric chronic diseases (p > 0.05, Table 1). At the first visit, none of the pediatric laboratory-confirmed COVID-19 patients and children and adolescents of the control group received the COVID-19 vaccine.

**Table 1:** Demographic, socio-economic, anthropometric, and laboratory data, previous pediatric chronic conditions in pediatric laboratory-confirmed Coronavirus Disease 2019 (COVID-19) at first visit compared to children and adolescents without laboratory-confirmed COVID-19 (control group).

Variables	Pediatric laboratory confirmed COVID-19 at first visit (n = 53)	Controls without COVID-19 (n = 52)	p
**Demographic data**			
Age, years	14.65 (8‒18)	14.84 (8‒18)	0.96
Time between COVID-19 diagnosis and follow-up, months	4.4 (0.8‒10.7)	‒	‒
Male sex	22 (42)	21 (40)	0.91
Ethnicity			
Caucasian	30 (57)	25 (48)	0.57
African Latin American	21 (49)	25 (48)	
Asian	1 (2)	0 (0)	
Others/unknown	1 (2)	2 (4)	
**Socio-economic data**			
Social assistance program	14/26 (54)	16/37 (43)	0.41
Family minimum wages/month	2 (0.2‒10)	2 (0‒8)	0.66
Household members in the residence, number	4 (2‒6)	4(2‒8)	0.69
**Anthropometric data**			
Body mass index, kg/m^2^	20.2 (14.5‒39.5)	19.6 (13.8‒34.9)	0.60
Obese	7/48 (15)	4/37 (11)	0.75
Overweight	10/48 (21)	7/37 (19)	1.00
**Laboratory data**			
Fasting blood glucose, mg/dL	79 (59‒291)	79 (63‒117)	1.00
Fasting blood glucose ≥ 126 mg/dL	1 (2)	0 (0)	1.00
A1C, %	5 (4.1‒6.8)	5 (3.7‒5.9)	1.00
A1C ≥ 6.5%	1 (2)	0 (0)	1.00
**Previous pediatric chronic diseases**	47 (89)	47 (90)	1.00
Previous diabetes mellitus	1 (2)	1 (2)	1.00
Immunosuppressive diseases	44 (83)	40 (77)	0.47
Transplantation	5 (9)	9 (17)	0.26
Neoplasia	7 (13)	3 (6)	0.32
Chronic kidney disease	3 (6)	2 (4)	1.00
Autoimmune diseases	14 (26)	9 (17)	0.37
Others chronic diseases	24 (45)	30 (58)	0.24
**Pediatric COVID-19 classification**			
Mild	41 (78)	‒	‒
Moderate	6 (11)	‒	‒
Severe and critical	6 (11)	‒	‒
**New-onset type 1 diabetes mellitus after COVID-19**	0/53 (2)	0/51 (0)	1.00
**Other new autoimmune conditions after COVID-19**	0/53 (2)	0/51 (0)	1.00

Results are shown in n (%), median (minimum value and maximum value), A1C, Glycated Hemoglobin.

Based on this evidence, we conducted an assessment of new-onset diabetes mellitus diagnoses in our patients following SARS-CoV-2 infection. To achieve this, we systematically analyzed fasting blood glucose and glycated hemoglobin (A1C) tests during both the first and second follow-up visits after SARS-CoV-2 infection in a cohort of 53 symptomatic pediatric patients with laboratory-confirmed COVID-19. The median duration between SARS-CoV-2 infection and the subsequent longitudinal follow-up analysis was 4.4 months during the first visit (ranging from 0.8 to 10.7 months) and 12.35 months during the second visit (ranging from 9.3 to 22.5 months).

These laboratory examinations were also conducted on a control group consisting of 52 children and adolescents. Members of this control group tested negative for SARS-CoV-2 based on both real-time reverse transcription-polymerase chain reaction (real-time RT-PCR) and antibody-based SARS-CoV-2 analysis. The control group was meticulously matched with the pediatric patients who had laboratory-confirmed COVID-19 in terms of age, gender, ethnicity, socioeconomic background, anthropometric data, and preexisting pediatric chronic diseases. Importantly, at the time of the first visit, none of the pediatric patients with laboratory-confirmed COVID-19 or the children and adolescents in the control group had received the COVID-19 vaccine (p > 0.05, Table 1).

Preexisting diabetes mellitus before COVID-19 diagnosis was observed in 1/53 (2%) of pediatric laboratory-confirmed COVID-19 patients and 1/52 (2%) in the control group (p = 1.000) (Table 1), with worsening diabetes mellitus symptoms after SARS-CoV-2 infection diagnosis in one patient.

Importantly, new-onset diabetes mellitus was not observed in both pediatric laboratory-confirmed COVID-19 patients (n = 0/53) and n = 0/52 (0%) in the control group at the first visit (p = 1.000, Table 1).

Crucially, it's worth noting that there were no cases of new-onset diabetes mellitus observed among both the pediatric patients with laboratory-confirmed COVID-19 (n = 0/53) and the control group (n = 0/52, 0%) during the initial visit (p = 1.000, as shown in Table 1). Furthermore, the absence of new-onset diabetes mellitus persisted during the second visit for the pediatric patients with laboratory-confirmed COVID-19 (n = 0/43, 0%), as assessed according to the recent diagnostic criteria for diabetes mellitus established by the American Diabetes Association (ADA).^6^ Table 2 presents comparable laboratory data for pediatric patients with preexisting chronic conditions who had laboratory-confirmed COVID-19 during both the initial and subsequent visits (p > 0.05).

**Table 2:** Comparisons between laboratory data of previous pediatric chronic conditions patients with laboratory-confirmed Coronavirus Disease 2019 (COVID-19) at first visit and second visit.

Variables	Pediatric laboratory confirmed COVID-19 at first visit (n = 53)	Pediatric laboratory confirmed COVID-19 at second visit (n = 43)	p
**Laboratory data**			
Fasting blood glucose, mg/dL	79 (59‒291)	79 (63‒138)	1.00
Fasting blood glucose ≥126 mg/dL	1 (2)	1 (2)	1.00
A1C, %	5 (4.1‒6.8)	5 (4.2‒7)	1.00
A1C ≥ 6.5%	1 (2)	1 (2)	1.00

Results are shown in n (%), median (minimum value and maximum value), A1C, Glycated Hemoglobin.

Notably, only a single patient displayed a sustained increase in A1C levels during both the initial and subsequent visits following SARS-CoV-2 infection, yet without confirmation of diabetes mellitus. This patient, a 17-year-old male, had a medical history significant for homozygous sickle cell disease and Moyamoya vasculopathy with a prior stroke presentation. He tested positive for COVID-19 in 2020 and was admitted to our institution for chronic transfusion therapy as a preventive measure against further strokes. Remarkably, he remained asymptomatic throughout his hospitalization, although he had reported symptoms including cough, sore throat, dysgeusia, anosmia, and chest pain ten days before seeking medical attention. His last transfusion had occurred two months prior to the episode, a gap attributed to pandemic-related challenges. Hemoglobin electrophoresis conducted before transfusion indicated 50.3% of S hemoglobin.

Upon hospital admission, the patient was initially asymptomatic, displaying no fever, nausea, vomiting, polyuria, polydipsia, weight loss, tiredness, diarrhea, or dyspnea. His physical examination revealed no abnormalities, with oxygen saturation and chest radiography within normal ranges. Notably, while a real-time RT-PCR test for SARS-CoV-2 returned a negative result, antibody-based SARS-CoV-2 analysis yielded a positive outcome.

After four months following the COVID-19 diagnosis, the patient remained asymptomatic. His body mass index was recorded at 22.4 kg/m^2^ (64^th^ percentile for his age), with an elevated A1C level of 6.5%, alongside normal fasting plasma glucose of 85 mg/dL and a standard C-peptide level of 3.98 ng/mL (within the range of 1.1‒4.4). Additionally, markers of type 1 diabetes mellitus auto-immunity, including Islet Antigen 2 (IA-2) autoantibody at 10 U/mL and glutamic acid decarboxylase antibody at 5 IU/mL, were within normal limits.

Upon reaching the 12-month mark following the COVID-19 diagnosis, the patient remained asymptomatic, without experiencing polyuria, polydipsia, weight loss, or tiredness. His body mass index was measured at 21.72 kg/m^2^ (67^th^ percentile for his age), accompanied by an elevated A1C level of 6.6%. Further laboratory results showed normal fasting plasma glucose (77 mg/dL), normal C-peptide (1.88 ng/mL), and normal postprandial blood glucose (122 mg/dL). Therefore, it is important to note that only one patient, who had experienced mild COVID-19 symptoms, exhibited a sustained increase in A1C levels without presenting any signs or symptoms indicative of diabetes mellitus following SARS-CoV-2 infection. It is worth emphasizing that updated diagnostic criteria for diabetes mellitus, as per the ADA, discourage the use of A1C concentrations as the sole diagnostic criterion in patients with hemoglobinopathies.^6^ Indeed, it is likely that the presence of sickle cell trait and/or hemoglobin C may have influenced the reliability of the A1C test in this particular case.^7^

Significantly, a recent study assessed the risk of new diabetes mellitus diagnoses (type 1, type 2, or other diabetes mellitus) among children and adolescents under 18 years of age in the United States within one month of acute SARS-CoV-2 infection. This study found a notably higher incidence of diabetes among those with COVID-19 compared to those without (hazard ratio 1.31, 95% Confidence Interval 1.20‒1.44).^5^ Another report revealed that during the COVID-19 pandemic, incident cases of pediatric type 2 diabetes mellitus surpassed those of type 1 diabetes mellitus.^7^

The mechanism through which SARS-CoV-2 infection leads to new-onset pediatric diabetes mellitus remains unknown. It may be associated with direct damage to pancreatic islet cells expressing angiotensin-converting enzyme 2 receptors, stress-induced hyperglycemia due to cytokine storms, disturbances in glucose metabolism triggered by SARS-CoV-2 infection, or the progression of prediabetes to pediatric diabetes mellitus.^2^, ^3^, ^4^, ^5^^,^^8^

In our current study, pre-existing diabetes mellitus was not identified as a risk factor for the development of severe pediatric COVID-19 and related complications, which contrasts with previous observations.^2^ In conclusion, our research demonstrates that new-onset diabetes mellitus following SARS-CoV-2 infection did not occur within a long-term cohort of pediatric patients who had preexisting chronic conditions and were under our care at a tertiary hospital.

## Financial support

This study was supported by grants from Conselho Nacional de Desenvolvimento Científico e Tecnológico (CNPq 304984/2020-5 to CAS), Fundação de Amparo à Pesquisa do Estado de São Paulo (FAPESP 2015/03756-4 to CAS) and by Núcleo de Apoio à Pesquisa “Saúde da Criança e do Adolescente” da USP (NAP-CriAd) to CAS.

## Declaration of Competing Interest

The authors declare no conflicts of interest.
